# Development and Psychometric Validation of a Patient-Reported Outcome Measure for Arm Lymphedema: The LYMPH-Q Upper Extremity Module

**DOI:** 10.1245/s10434-021-09887-y

**Published:** 2021-07-05

**Authors:** Anne F. Klassen, Elena Tsangaris, Manraj N. Kaur, Lotte Poulsen, Louise M. Beelen, Amalie Lind Jacobsen, Mads Gustaf Jørgensen, Jens Ahm Sørensen, Dalibor Vasilic, Joseph Dayan, Babak Mehrara, Andrea L. Pusic

**Affiliations:** 1 McMaster University, Hamilton, ON Canada; 2 Brigham and Women’s Hospital, Harvard Medical School, Boston, MA USA; 3 Research Unit for Plastic Surgery, Odense University Hospital, Odense, Denmark; 4 University of Southern Denmark, Odense, Denmark; 5 Odense Explorative Patient Network, Odense, Denmark; 6 Department of Plastic, Reconstructive and Hand Surgery, ErasmusMC, Rotterdam, The Netherlands; 7 Memorial Sloan-Kettering Cancer Center, New York, USA

## Abstract

**Background:**

A multiphased mixed-methods study was performed to develop and validate a comprehensive patient-reported outcome measure (PROM) for arm lymphedema in women with breast cancer (i.e., the LYMPH-Q Upper Extremity Module).

**Methods:**

Qualitative interviews (January 2017 and June 2018) were performed with 15 women to elicit concepts specific to arm lymphedema after breast cancer treatment. Data were audio-recorded, transcribed, and coded. Scales were refined through cognitive interviews (October and Decemeber 2018) with 16 patients and input from 12 clinical experts. The scales were field-tested (October 2019 and January 2020) with an international sample of 3222 women in the United States and Denmark. Rasch measurement theory (RMT) analysis was used to examine reliability and validity.

**Results:**

The qualitative phase resulted in six independently functioning scales that measure arm symptoms, function, appearance, psychological function, and satisfaction with information and with arm sleeves. In the RMT analysis, all items in each scale had ordered thresholds and nonsignificant chi-square *p* values. For all the scales, the reliability statistics with and without extremes for the Person Separation Index were 0.80 or higher, Cronbach’s alpha was 0.89 or higher, and the Intraclass Correlation Coefficients were 0.92 or higher. Lower (worse) scores on the LYMPH-Q Upper Extremity scales were associated with reporting of more severe arm swelling, an arm problem caused by cancer and/or its treatment, and wearing of an arm sleeve in the past 12 months.

**Conclusions:**

The LYMPH-Q Upper Extremity Module can be used to measure outcomes that matter to women with upper extremity lymphedema. This new PROM was designed using a modern psychometric approach and, as such, can be used in research and in clinical care.

Breast cancer treatment is the most common cause of upper extremity lymphedema in Western countries.^1^ Risk factors for the development of arm lymphedema are axillary lymph node dissection (ALND), sentinel lymph node biopsy (SLNB), and radiation therapy of the axilla, or a combination of these therapeutic methods.

The overall incidence of breast cancer-related arm lymphedema has ranged between 14 and 21%.^1^–^3^ Findings from a prospective cohort study of 2171 women investigating time course and incidence of breast cancer-related lymphedema identified that patients receiving ALND with radiation therapy were at a greater risk for the development of lymphedema (31.2 %) than those with ALND alone (24.6 %) or with SLNB plus radiation therapy (12.2 %).^2^ Furthermore, early onset of lymphedema at less than 12 months postoperatively was associated with having ALND, whereas onset at 12 months or later was associated with having radiation therapy.^2^

Arm lymphedema is a debilitating diagnosis that may impose a significant detriment to a patient’s health-related quality of life (HRQOL) due to symptoms (e.g., swelling, pain, infection) and reduced arm function.^4^,^5^ The rapidly emerging field of lymphedema research has had little consensus on the most suitable metric for measuring outcomes. Clinicians commonly use limb volume and circumference, but these metrics do not capture the HRQOL burden of arm lymphedema from the patient perspective.^6^,^7^

To better understand and measure outcomes that matter to patients with arm lymphedema, a valid and reliable patient-reported outcome measure (PROM) is needed. Given that arm lymphedema affects how patients function and feel, information captured through the use of a PROM may be a better indicator of disease than traditional clinical metrics.^6^

Currently, 14 lymphedema-specific PROMs have been used to measure upper extremity lymphedema outcomes from the patient perspective.^8^ A systematic literature review identified that 13 of the 14 PROMs were developed with limited input from patients.^8^ Patient involvement in the development of a PROM is considered crucial to ensuring that its content is comprehensive and relevant to patients (i.e., content validity).^9^,^10^ The systematic literature review also identified that the quality of each PROM was low to moderate in terms of meeting the criteria for reliability and validity as set out in the COnsensus‐based Standards for the selection of health Measurement Instruments (COSMIN).^11^

To address the shortcomings in existing PROMs for upper extremity lymphedema, our team developed the LYMPH-Q Upper Extremity Module. This PROM was developed to complement the BREAST-Q that we previously developed to measure HRQOL and patient satisfaction among women with breast surgery.^12^ This study aimed to describe the development and psychometric validation of the LYMPH-Q Upper Extremity Module.

## Methods

Best practice guidelines for the development of a PROM were used to guide this multi-phased mixed-methods study.^10^,^13^–^17^ Phase 1 involved qualitative patient interviews to elicit concepts. Interpretive description was used to inform the qualitative approach.^18^ Subsequently, cognitive interviews with patients and expert input were used to refine the new scales content. In phase 2 (quantitative), a field-test was performed and Rasch measurement theory (RMT)^19^,^20^ analysis was used for item reduction and to examine the psychometric properties of each scale.

### Research Ethics

For phase 1, approval was obtained from the Research Ethics Boards at McMaster University (Hamilton, ON, Canada), Toronto General Hospital (TGH) (Toronto, ON, Canada), Memorial Sloan Kettering Cancer Center (MSK) (New York, NY, USA), and Brigham and Women’s Hospital (BWH) (Boston, MA, USA). In Denmark, the study was reported to and approved by the Region of Southern Denmark and included on the list of Health Research for data protection safety.

Written consent was obtained from all the participants before each qualitative and cognitive interview. The participants in Canada and the United States were sent a $50 (CAD, USD) gift card to thank them for their participation.

For phase 2, in the United States, approval was obtained from BWH and the Scientific Advisory Committee of the Love Research Army (LRA; formerly known as the Army of Women), an online non-profit community started by the Dr. Susan Love Research Foundation in 2008 that connects breast cancer researchers to women with and without breast cancer.^21^ An email describing the study aims was sent to LRA members. Completion of the study questionnaire implied consent.

For Denmark, phase 2 of the study was approved by the Region of Southern Denmark and included on the list of Health Research for data protection safety. Ethics approval from the Regional Committee on Health Research Ethics was not required because the study involved completion of a questionnaire. An email invitation was sent to the electronic secure mailbox (Eboks) of potential participants. Informed consent to take part in the study was obtained electronically in REDCap.

### Phase 1: Qualitative Interview

#### Sample and Recruitment

Women who were 18 years of age or older with a breast cancer diagnosis and fluent in English were invited to participate in a qualitative interview as part of a larger study to develop new scales for the BREAST-Q. A purposive sampling approach was used to ensure that participants varied by age and breast cancer stage (stages 0–4), as well as by surgical (i.e., breast-conserving therapy, mastectomy with/without reconstruction) and nonsurgical (i.e., adjuvant or neoadjuvant) breast cancer treatments. Data from the subset of women in the sample who reported arm lymphedema were used to develop the LYMPH-Q Upper Extremity Module.

Health care professionals described the study to potential participants in clinics or by telephone. Permission was obtained to share contact information with the research team. Interviews were scheduled and took place by phone or face-to-face at a time that was convenient to each participant.

#### Concept Elicitation

Interviews were performed by experienced qualitative researchers who followed a semi-structured interview guide with open-ended questions. The participants were asked to discuss how lymphedema and its treatment influenced their physical, psychological, and social well-being, as well as their overall HRQOL. The interviews were audio-recorded and transcribed verbatim.

To establish rigor, data collection and analysis took place concurrently so that new concepts elicited from participants could be added to the interview guide. Furthermore, the interviews were coded by two coders independently, and regular team meetings were used to review coding. Multiple levels of codes (top-level domains and themes) were applied to the text. Codes were created inductively through the generation of new codes and deductively through the application of relevant codes from the BREAST-Q conceptual framework.^12^ Recruitment continued until redundancy of concepts elicited through the interviews was achieved.

Participant quotes and associated codes were transferred from Microsoft Office Word to Excel for further refinement of themes and subthemes using constant comparison. In Excel, an item pool was developed for use in scale development. Scales covered key concepts elicited from the participants. Each scale was given instructions, a time frame for answering, and a set of response options.

#### Scale Development and Refinement

Patient and expert input was used to establish content validity of the LYMPH-Q Upper Extremity Module. A semi-structured cognitive interview guide was used with questions and probes to determine whether the content of each scale (i.e., instructions, recall period, item set, response options) was comprehensive, relevant, and comprehensible.^15^ Participants were asked to suggest missing concepts. Women with breast cancer-related lymphedema from Canada, the United States, and Denmark 18 years of age or older who could speak and read in English or Danish, were invited to participate in the cognitive interviews, which used the think-aloud approach.^22^–^24^ Interviews and analyses were performed by skilled qualitative researchers and took place in rounds to enable the refinement of scale content between rounds.

Experts known to our team who treat patients with arm lymphedema were invited to provide feedback on the comprehensiveness and relevance of the LYMPH-Q Upper Extremity Module content. An email invitation was sent by a member of the research team with an attached PDF copy of the lymphedema scales. A reminder email was sent after 1-week. Experts were asked to provide written feedback via email and to add missing concepts. The input from the experts was analyzed descriptively by two researchers with the results used to refine the scales.

Elsewhere we describe the methods and results of a linguistic validation study to translate the LYMPH-Q Upper Extremity Module into Danish.^25^ The scales were translated into Danish according to the International Society for Pharmacoeconomics and Outcomes Research^26^ and World Health Organization^27^ guidelines. Feedback from patients and experts provided additional evidence of the scales’ content validity.

### Phase 2: Field-Test Study

#### Sample and Recruitment

The analysis included data from two samples as follows:

1. Love Research Army

The LRA study was performed as part of a larger study to develop new scales for the BREAST-Q. The study was open to women 18 years of age or older with a diagnosis of breast cancer who could read English. The LRA members were sent an electronic recruitment email (e-blast) containing a description of the study and the eligibility criteria. Women who agreed to participate were directed to a REDCap survey^28^ designed by our team and hosted at BWH.

The REDCap survey included demographic and clinical questions, new BREAST-Q scales and the LYMPH-Q Upper Extremity Module. Targeted clinical questions and branching logic were used to ensure that only women with arm lymphedema completed the LYMPH-Q Upper Extremity scales. The LRA participants were invited to take part in a test-retest (TRT) study. Those who provided their email were automatically sent a URL link 3 weeks after the initial survey, with one reminder sent after 2 days.

2. Danish National Health Data Authority

In Denmark, a list of all patients 18 years of age or older with a diagnosis of both breast cancer and arm lymphedema in the past 12 years was obtained from the Danish National Health Data Authority. An invitation with written information about the study and a REDCap public link to the survey was sent to patients’ Eboks. The REDCap database was hosted by the Open Patient Data Explorative Network.^29^ Patients were invited to use the URL provided to access and complete clinical and demographic questions as well as the LYMPH-Q Upper Extremity Module. Two reminders were sent 7 and 14 days after the initial invitation.

### Data Analysis

In this study, RMT analysis was performed using RUMM2030 software and the unrestricted Rasch model for polytomous data (RUMM version 2030; RUMM Laboratory Pty Ltd., Duncraig, Western Australia, 1998-14). The RMT analysis involved a series of diagnostic tests, described in detail elsewhere.^30^ Briefly, a set of statistical and graphic tests, were used to identify items and scales that did not work as hypothesized.^19^ Scales that work have a set of items that line up to map out a single continuum. RMT analysis uses the chi-square statistic to examine both item fit and the overall model fit. Because this test is highly sensitive to sample size, we adjusted the sample to 500. We also applied Bonferroni corrections to account for multiple comparisons.

#### Ordering of Item Thresholds

Threshold maps were examined to determine whether response options (e.g., very dissatisfied, somewhat dissatisfied, somewhat satisfied, very satisfied) were used appropriately. Disordered thresholds indicate problems in comprehension, or that the response options do not work as intended.

#### Item Fit

We examined individual item fit and overall fit of the data to the Rasch model.^31^,^32^ Indicators of fit were inspected and interpreted together. Item fit was evaluated statistically by whether fit residuals were within – 2.5 and + 2.5 and had nonsignificant chi-square values after Bonferroni adjustment. Fit residuals also were inspected graphically to determine whether item characteristic curves showed agreement between observed and expected scores.^31^,^32^

#### Local Dependency

We examined residual correlations to identify their influence on the Person Separation Index (PSI, reliability). Any pairs of items with a residual correlation of 0.30 or higher were included in a subtest to determine their impact on scale reliability.^33^–^35^

#### Targeting

Scales should measure the construct as experienced by the sample. We examined graphic displays (person-item threshold distributions) of item and person spread to determine whether these overlapped. We also computed the proportion of the sample that scored outside the range of measurement.

#### Differential Item Function (DIF)

We examined whether subgroups in the sample responded differently to items in a scale despite having similar level of the construct measured. DIF was examined for dataset (USA and Danish) and age group (18 to 49, 50 to 59, 60 to 69, ≥ 70 years). For each variable, we performed DIF three times with random samples drawn to match the smallest subgroup. The DIF then was performed with and without adjustment of the overall sample in the analysis to 500. Any items that evidenced significant DIF in the unadjusted analysis were split on the sample characteristic. The person locations based on the original and split analyses were correlated to determine whether the DIF had any impact on scale scoring.^32^

#### Reliability

The PSI and Cronbach alpha^36^ reliability coefficients were determined within RUMM2030. Values of 0.70 or higher were considered acceptable.^37^

From the final models for each scale, the Rasch scores were obtained and transformed from 0 (worst) to 100 (best). The following statistical tests were performed in SPSS version 26.0 (IBM Corporation, Armonk NY, USA for Windows/Apple Mac).

1. *Test-Retest Reliability.* We used the transformed scores of 0 to 100 to examine test-retest reliability. The participants were asked if anything had changed with their health or in their life since they completed the questionnaire (response options: yes, no). Those who said yes were excluded from the TRT analyses. We computed two-way random Intraclass Correlation Coefficients (ICCs) with the test-retest data. ICC values greater than 0.70 were considered acceptable.^38^

2. *Data Quality.* We examined scale-level missing data and the proportion of patients to score at the floor and ceiling.

3. *Construct Validity.* We examined the normality of the data by examining kurtosis and skewness.^39^ Data that exceeded ± 2.0 were examined using nonparametric statistics.^39^ We tested the following hypotheses. First, we expected that correlations between the scales measuring similar, related but dissimilar, and unrelated constructs would meet the COSMIN guidelines for construct validity (i.e., correlations should be ≥0.50 for similar constructs, 0.30 to 0.50 for related but dissimilar constructs, and < 0.30 for unrelated constructs).^11^ Second, we expected that participants’ scale scores would be incrementally associated with severity (none, mild, moderate, severe) of self-reported arm swelling. Third, we expected that participants’ scale scores would be incrementally associated with having a self-reported arm problem (none, minor, major) as a result of breast cancer, treatment, or both. Finally, we expected that scale scores would be lower for women who reported that they wore a compression sleeve in the past 12 months compared with those who did not.

## Results

### Phase 1: Qualitative Phase

Data collection took place between January 2017 and June 2018. Qualitative interviews were performed with 58 patients as part of the larger BREAST-Q study. Data from 15 participants with arm lymphedema were used to develop the LYMPH-Q Upper Extremity Module scales. Those with arm lymphedema were mainly 40 to 74 years of age. The participants included 13 white patients and 10 married patients. Most of the participants had a mastectomy (*n* = 10) and a history of combination treatment with chemotherapy, radiotherapy, or endocrine therapy (*n* = 7).

Analysis resulted in the development of a framework of concepts important in arm lymphedema. The framework included top-level domains with two or more of the following major themes: arm appearance (body image, characteristic, clothing), physical (function, symptoms), psychological (distress, impact), social (support, function, relationships), and experience of care (lymphedema information, arm sleeve). The item pool was used to develop content for five LYMPH-Q Upper Extremity Module scales as follows: function, symptoms, appearance, life impact, and information. Each scale was assigned instructions, a time frame for responding, and four response options that measured severity (symptoms, life impact), bother (appearance), difficulty (function), and satisfaction (information).

To establish content validity further, we performed 16 cognitive interviews of patients with breast cancer who had arm lymphedema. Interviews took place in three rounds between October and December 2018. Round 1 included two U.S. participants; round 2 included 10 Danish participants; and round 3 included four U.S. participants. The sample included women 38 to 74 years of age who were mainly white (*n* = 16) and married (*n* = 11). Most of the participants had a mastectomy (*n* = 10), ALND (*n* = 14) and a history of a combination of chemotherapy, radiotherapy, and endocrine therapy (*n* = 11).

Feedback was obtained from 12 of 22 invited multi-disciplinary experts after round 2 (response rate, 55 %). The experts represented four countries (Canada, Denmark, Poland, United Kingdom) and included eight plastic surgeons, two breast surgeons, a medical oncologist, and a nurse practitioner.

In round 1, the participants reviewed 57 items in five scales (symptoms, function, appearance, life impact, and information). Two new scales (psychological, arm sleeves) were added after round 1 participant feedback. The psychological and arm sleeve scales measured whether lymphedema affects how participants feel (response options: always, often, sometimes, never) and satisfaction with the arm sleeve (response options: very dissatisfied, somewhat dissatisfied, somewhat satisfied, very satisfied), respectively. The final set of items was tested in round 3, which resulted in a total of 110 items in the following scales finalized for the field-test: symptoms (*n* = 20 items), function (*n* = 19 items), appearance (*n* = 14 items), life impact (*n* =11 items), psychological (*n* = 19 items), information (*n* = 13 items), and arm sleeve (*n* = 14 items).

The LYMPH-Q Upper Extremity Module was translated into Danish.^25^ The scales were reviewed by an expert panel consisting of the primary investigator leading the translation process, two professional bilingual translators, a breast surgeon/plastic surgeon, a physiotherapist specialized in lymphedema treatment, a medical doctor specialized in lymphedema research, and a medical doctor specialized in PROM research. This was followed by cognitive debriefing interviews with 10 women who had arm lymphedema. The feedback received by the patients and the expert panel confirmed that the scales were comprehensive and comprehensible and included highly relevant questions.

### Phase 2: Quantitative Phase

Field-test data were collected between October 2019 and January 2020. A total of 1717 LRA members opened the REDCap link and self-selected themselves to be eligible for the study. Among these members, 364 had a diagnosis of lymphedema and completed at least one of the lymphedema scales. Of the 364 participants, 79 also provided data for the TRT.

In Denmark, 8139 women with breast cancer and arm lymphedema were identified. Of these women, 6850 used Eboks and were invited to participate. Responses were obtained from 3945 women (57.6 %). Of these women, 1087 were excluded from the study (426 declined to participate, 298 did not have lymphedema, 363 completed only the clinical/demographic information). After these exclusions, 2858 Danish participants were included in the analysis. Sample characteristics for the combined sample of 3222 participants are presented in Table 1.

**Table 1:** Characteristics of the 3222 participants in the field-test sample

	*n*	%
*Country*		
Denmark	2858	88.7
USA	364	11.3
*Age group (years)*		
≤49	322	10.0
50–59	854	26.5
60–69	1037	32.2
≥70	1009	31.3
*BMI (kg/m*^2^)		
Under/normal weight (<25)	1226	38.0
Overweight (25–29)	1107	34.4
Obese (≥30)	877	27.2
Missing	12	0.4
*Ethnicity*		
White	2806	87.1
Other	416	12.9
*Relationship status*		
Married/common-law	2349	73.0
Separated/divorced	235	7.3
Widowed	285	8.8
Single, never married	336	10.4
Other	17	0.5
*Education status*		
Some high school	313	9.7
Completed high school	360	11.2
Some college, trade, or university	714	22.2
Completed college, trade, or university	1245	38.6
Some Masters or Doctoral	331	10.3
Completed Masters or Doctoral	164	5.1
Other	95	2.9
*Employment status*		
Retired	1630	50.6
Working full-time	726	22.5
Working part-time	499	15.5
Other	367	11.4
*Treatment for breast cancer*		
None	77	2.4
Chemotherapy	2447	76.0
Radiation therapy	2912	90.4
Anti-estrogen therapy	2303	71.5
Targetted therapy	593	18.4
*Arm swelling*		
None	418	13.0
Mild	1346	41.8
Moderate	1070	33.2
Severe	356	11.0
Missing	32	1.0
*Lymphedema laterality*		
Unilateral	3156	98.0
Bilateral	66	2.0
*Arm problem as a result of breast cancer and/or treatments*		
None	335	10.4
Minor	2108	65.4
Major	779	24.2
*Time since lymphedema diagnosis (years)*		
≤4	998	31.0
5**–**9	1183	36.7
≥10	1041	32.3
*Compression sleeve worn in the past 12 months to reduce or prevent swelling?*		
Yes	2282	70.8
No	940	29.2
*Bothered by how arm(s) look overall?*		
Not at all	1154	35.8
A little	1049	32.6
Moderately	604	18.7
Extremely	353	11.0
Missing	62	1.9

SN, sentinel node biopsy; ALND, axillary lymph node dissection

The RMT analysis led to a reduction of items from 110 to 68. Items were dropped due to either poor fit to the Rasch model or redundant content. All 68 items had properly ordered thresholds (Appendix 1) and nonsignificant chi-square *p* values after Bonferroni adjustment. Data fit the Rasch model for all six scales, with nonsignificant *p* values (Appendix 2). Item fit was within ± 2.5 for 27 of the 68 items. Scale level findings for the six scales that formed the item-reduced version of the LYMPH-Q Upper Extremity Module are shown in Appendix 2, and item fit statistics are shown in Appendix 3. The proportion of the sample that scored on each scale is shown in Appendix 2. All but one scale (psychological) had at least 80 % of participants’ scores within the scale’s measurement. Targeting can be seen graphically in Appendix 1, which shows the person measurement and item locations for each scale.

Differential item function was evident for 31 items in the unadjusted analysis that compared the Danish and U.S. datasets and for 14 items by age group (Appendix 3). In the adjusted analysis, DIF was evident for 26 items by dataset and for 3 items by age group (Appendix 3). When the items that evidenced DIF in the unadjusted analysis were split by the relevant participant characteristics, Spearman correlations between the original and split-person locations indicated that DIF had a negligible impact (*r* ≥ 0.991 for all correlations).

The PSI values were 0.80 or higher (with and without extremes), and the Cronbach alpha values were 0.89 or higher (with and without extremes) (Appendix 2). One pair of items in each of the symptoms (swelling, heavy), function (hold phone, hold book), and appearance (photos, noticeable) scales had residuals that correlated greater than 0.30. A subtest performed on these three item pairs showed the impact on the PSI values to be marginal, with a maximum drop in PSI of less than 0.01 with and without extremes.

Test-retest data were provided by 79 of the participants. Five of the participants reported a change in their health or life since completing the scales and were excluded. Appendix 2 shows the ICC values with 95 % confidence intervals. The ICC values for the six scales was 0.92 or higher. The scale-level missing data value was low (≤ 1.4 %, see Appendix 2). Floor effects were low (≤ 4.3 %), and ceiling effects ranged from 4.1 % (symptoms) to 22.7 % (psychological) (Appendix 2). The mean grade reading levels for the items in each scale were between 2.5 (symptoms, sleeve) and 15.6 (psychological), and the grade reading levels for the instructions ranged from 3.7 (psychological) to 14.1 (information).

Table 2 shows the Pearson correlations between the LYMPH-Q Upper Extremity Module scales. As hypothesized, the correlations between the scores on the four outcome scales were stronger with each other than with the two satisfaction scales. The correlations between the four outcome scales all met the level of >0.50 for related measures. The only correlations not in accordance with our hypothesized values, as per the COSMIN guidelines for construct validity, were the correlations between the arm sleeves scale and the symptoms, function, and psychological scales, which were higher than predicted.

**Table 2:** Pearson correlations between the LYMPH-Q upper extremity module scales

LYMPH-Q scales		*R*	*n*	Hypothesized relationship	Meets criteria
Symptoms	Function	0.774^a^	3213	Similar	Yes
	Appearance	0.591^a^	3208	Similar	Yes
	Psychological	0.623^a^	3194	Similar	Yes
	Information	0.207^a^	1756	Unrelated	Yes
	Arm sleeve	0.373^a^	2257	Unrelated	No
Function	Symptoms	0.774^a^	3213	Similar	Yes
	Appearance	0.504^a^	3206	Similar	Yes
	Psychological	0.575^a^	3192	Similar	Yes
	Information	0.174^a^	1753	Unrelated	Yes
	Arm sleeve	0.333^a^	2255	Unrelated	No
Appearance	Symptoms	0.591^a^	3208	Similar	Yes
	Function	0.504^a^	3206	Similar	Yes
	Psychological	0.562^a^	3191	Similar	Yes
	Information	0.222^a^	1753	Unrelated	Yes
	Arm sleeve	0.411^a^	2254	Related but dissimilar	Yes
Psychological	Symptoms	0.623^a^	3194	Similar	Yes
	Function	0.575^a^	3192	Similar	Yes
	Appearance	0.562^a^	3191	Similar	Yes
	Information	0.246^a^	1755	Related but dissimilar	Yes
	Arm sleeve	0.422^a^	2253	Unrelated	No
Information	Symptoms	0.207^a^	1756	Unrelated	Yes
	Function	0.174^a^	1753	Unrelated	Yes
	Appearance	0.222^a^	1753	Unrelated	Yes
	Psychological	0.246^a^	1755	Unrelated	Yes
	Arm sleeve	0.361^a^	1347	Related but dissimilar	Yes
Arm sleeve	Symptoms	0.373^a^	2257	Unrelated	No
	Function	0.333^a^	2255	Unrelated	No
	Appearance	0.411^a^	2254	Related but dissimilar	Yes
	Psychological	0.422^a^	2253	Unrelated	No
	Information	0.361^a^	1347	Related but dissimilar	Yes

^a^*p* ≤ 0.001; criteria: similar constructs, ≥ 0.50; related but dissimilar constructs, 0.30–0.50; unrelated constructs, <0.30

**Fig. 1:**
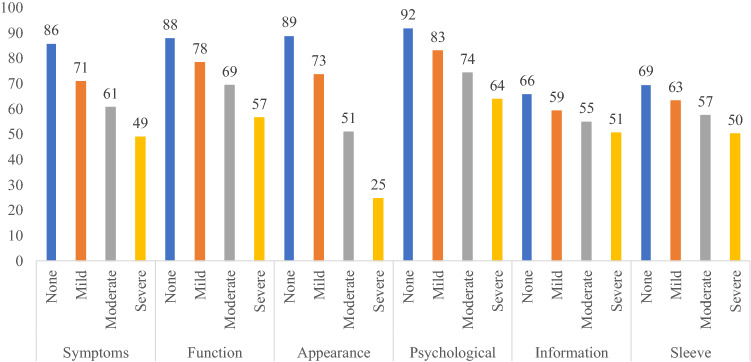
Mean scores for LYMPH-Q scales based on self-reported severity of arm swelling

**Fig. 2:**
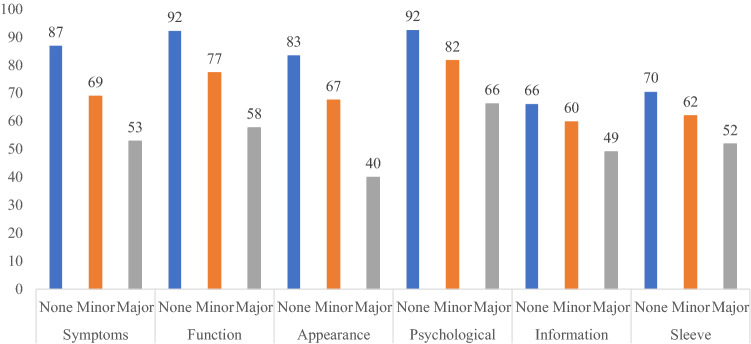
Mean scores for LYMPH-Q scales based on having a problem with the arm(s) as a result of breast cancer and/or its treatment

**Fig. 3:**
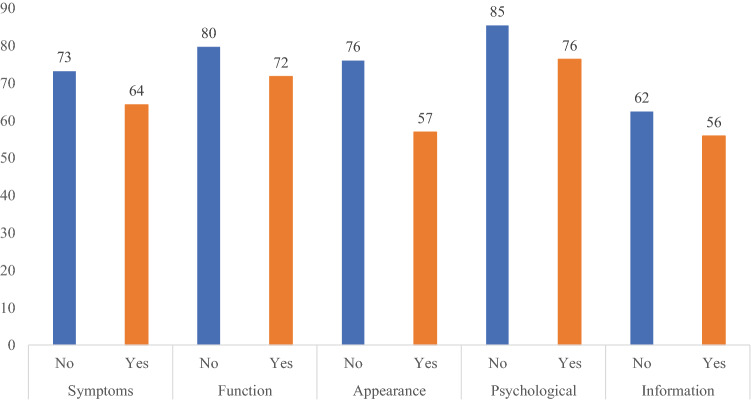
Mean scores for LYMPH-Q scales based on whether the participant wore a compression sleeve in the past 12 months

Consistent with our hypotheses, increased severity of arm swelling (Fig. 1), reporting of an arm problem caused by cancer or cancer treatments (Fig. 2), and wearing of a compression sleeve to reduce or prevent swelling in the past 12 months (Fig. 3) all were meaningfully associated with worse outcomes in all six LYMPH-Q Upper Extremity Module scales. Differences between scale scores by subgroups were statistically significant (*p* < 0.001) for all the scales in these three hypotheses. The characteristics of the subgroups for these tests of construct validity can be found in Appendix 4a–c.

## Discussion

The LYMPH-Q Upper Extremity Module provides clinicians and researchers with a rigorously developed PROM that can be used to measure outcomes of breast cancer-related arm lymphedema. Given the high prevalence of arm lymphedema and its significant impact on HRQOL, this new PROM represents an important addition to the literature.

The lack of an upper extremity lymphedema PROM developed with patient input has impeded advancements in the field of lymphedema research and treatment. Whereas an increasing number of studies have used HRQOL as the primary outcome in lymphedema research,^7^,^40^–^42^ recent literature reviews by Coriddi et al.^42^ and Beelen et al.^8^ have highlighted the frequent use of ad hoc instruments and generic PROMs to assess HRQOL. Ad hoc questionnaires are surveys, often composed for a specific study, with unknown psychometric properties. Generic PROMs are those designed for use with any patient population, which therefore do not ask about lymphedema-specific concerns. Although generic PROMs can facilitate comparison of outcomes across disease groups, such PROMs may not detect clinically important change after treatment for specific patient groups.^43^,^44^

For patients with lymphedema, no single objective measure adequately reflects the totality of a patient’s disability. Limb volume is a commonly used measure, but it can fluctuate throughout the day and can be manipulated with physiotherapy. In addition, this measurement does not account for other important concerns, such as recurrent cellulitis, physical disability, and psychological distress. Patient-reported outcomes in lymphedema used in conjunction with objective measures would provide a more complete picture of whether a therapeutic intervention has helped or not.

Due to the complex nature of lymphedema, greater fidelity in patient-reported outcome measurement is needed, which prompted the development of the LYMPH-Q Upper Extremity Module. The LYMPH-Q Upper Extremity Module was developed using a modern psychometric approach. Patient input ensured that the concepts most important to patients with lymphedema were identified and used to form the scales. The use of RMT analysis ensured that the scales provide interval-level measurement and are well-suited for use in individual patient care settings. The module can be used to evaluate the impact of new medical and surgical interventions for arm lymphedema, such as lymphovenous anastomosis and vascularized lymph node transplantation.

When choosing a PROM, high content validity, largely established through qualitative input from patients who have the condition of interest, is vital to measurement of change after an intervention. An important strength of our study was the careful qualitative research performed to ensure that the LYMPH-Q Upper Extremity Module had content validity for patients in three countries and was validated in two languages. Furthermore, evidence from the field-test study showed that the LYMPH-Q Upper Extremity Module worked the same by language and by age in the DIF analysis. These findings are important because they mean that the LYMPH-Q Upper Extremity Module provides a common metric with comparable scoring that can help to facilitate international research in lymphedema treatments.

Our study had several limitations. The initial qualitative sample did not include Danish participants, and the field-test sample did not include Canadians. However, for the Danish participants, we were able to ensure that the scales had content validity by including 10 women with arm lymphedema in the cognitive interviews performed to refine the scales and 10 additional women in the review of the Danish translation. Future research is needed to test the scales in a Canadian population.

To collect a large sample of data, we used an online survey, which can provide a large sample quickly at a low cost. Online surveys, however, do not reach participants with no Internet access and those who have access but are not active online. The majority of our participants were Danish and white, which limits applicability. Further validation studies could include a more diverse sample recruited from other countries.

Finally, we discovered a problem with the branching logic in the longitudinal setup within LRA REDCap for the test-retest reliability portion of the study. As a result, the LYMPH-Q Upper Extremity Module was sent 3 weeks after treatment instead of the planned 1 week after treatment. However, we believe 3 weeks still are a valid time for assessment of test-retest reliability.^45^ Because our research used a cross-sectional study design, testing the responsiveness of the scales was beyond the scope of this study. Future research is needed to examine the ability of the LYMPH-Q Upper Extremity Module scales to measure change and establish a minimal important difference.

## Conclusion

The LYMPH-Q Upper Extremity Module was created through a rigorous development process with an emphasis on qualitative input from patients and experts. It addresses an unmet need in the literature by providing a PROM for use in upper extremity lymphedema care and outcomes research with strong content and construct validity.

## Figures and Tables

**Figure:**
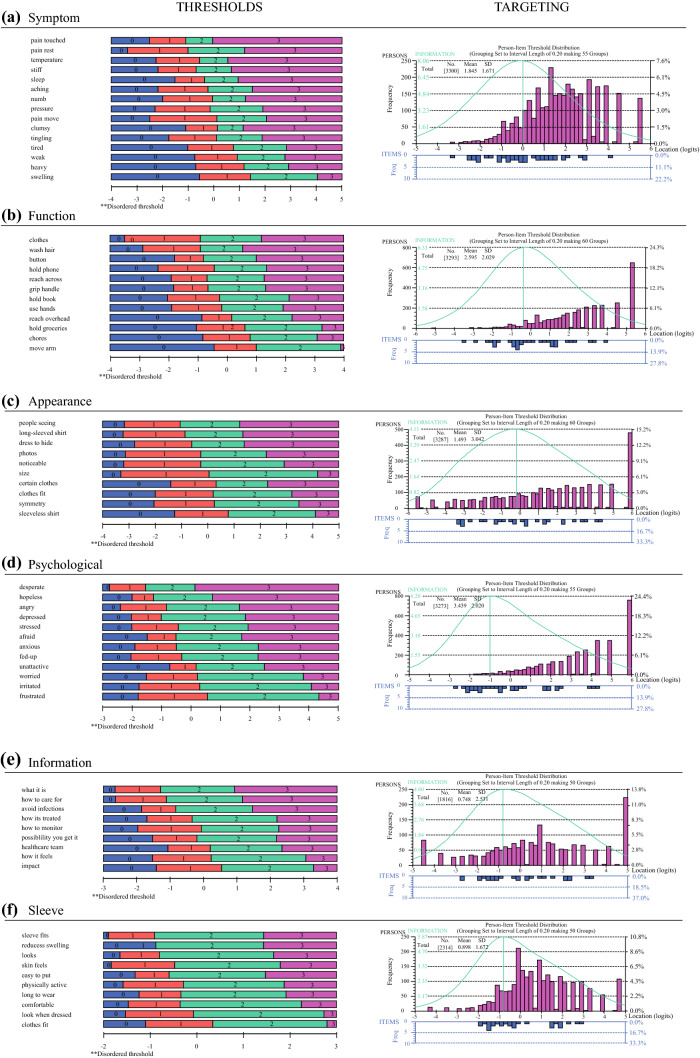
Threshold maps and person-item threshold distributions for each LYMPH-Q Upper Extremity scale from the RMT analysis

## Appendix 2

**Table:** Scale level results for the LYMPH-Q Upper Extremity Module

Scale	RMT analysis										ICC (95% CI)	Floor %	Ceiling %	Missing %
	# completed scale	# included in RMT	Scored on scale %	*χ*^2^	*DF*	*P* value	PSI +extr	PSI -extr	α +extr	α -extr				
Appearance	3287	2721	82.8	62.09	90	0.99	0.93	0.92	0.97	0.95	0.96 (0.94–0.98)	2.2	14.3	0.4
Psychological	3273	2505	76.5	99.27	96	0.39	0.80	0.83	0.93	0.91	0.94 (0.90–0.96)	0	22.7	0.8
Function	3293	2635	80.0	57.51	96	0.99	0.84	0.87	0.94	0.92	0.95 (0.92–0.97)	0.2	19.0	0.2
Symptoms	3300	3157	95.7	127.17	135	0.67	0.88	0.89	0.93	0.93	0.92 (0.87–0.95)	0	4.1	0.1
Arm sleeve	2314	2186	94.5	59.80	90	0.99	0.87	0.86	0.91	0.89	0.94 (0.89–0.96)	0.5	4.5	1.1
Information	1816	1504	82.8	83.89	72	0.16	0.91	0.90	0.95	0.92	0.92 (0.85–0.95)	4.3	11.8	1.4

*χ*^2^ = chi square; *df* = degrees of freedom; PSI + extr = Person Separation Index with extremes; PSI-extr = Person Separation Index without extremes; α+extr = Cronbach alpha with extremes; α-extr = Cronbach alpha without extremes; ICC = intraclass correlation coefficient; Floor = percent of participants scoring at the bottom of the scale; Ceiling = percent of participants scoring at the top of the scale

## Appendix 3

**Table:** RMT item level fit statistics and differential item function results

Scales	Item fit statistics							Differential item function*			
Item	Location	SE	Fit residual	*df*	*χ*^2^	*df*	Probability	Dataset		Age-group	
								Unadj	Adj	Unadj	Adj
*Symptoms*											
Pain touch	− 1.20	0.04	− 1.29	2880	3.90	9	0.92	No	No	No	No
Pain rest	− 1.05	0.03	− 4.41	2899	6.29	9	0.71	No	No	No	No
Temperature	− 0.75	0.03	2.17	2877	3.71	9	0.93	No	No	No	No
Stiff	− 0.72	0.03	− 2.03	2859	4.17	9	0.90	Yes	Yes	No	No
Sleeping	− 0.29	0.03	− 1.05	2893	2.85	9	0.97	Yes	Yes	No	No
Aching	− 0.29	0.03	− 7.90	2875	14.69	9	0.10	Yes	Yes	No	No
Numb	− 0.27	0.03	7.08	2864	12.11	9	0.21	No	No	No	No
Pressure	− 0.16	0.03	− 6.20	2872	10.86	9	0.29	No	No	No	No
Pain move	− 0.10	0.03	− 5.44	2905	8.95	9	0.44	No	No	No	No
Clumsy	0.07	0.03	5.07	2882	6.34	9	0.71	No	No	No	No
Tingling	0.09	0.03	1.36	2883	3.17	9	0.96	No	No	No	No
Tired	0.87	0.03	− 10.03	2884	18.41	9	0.03	Yes	Yes	Yes	No
Weak	1.00	0.03	− 1.00	2893	1.61	9	1.00	No	No	No	No
Heavy	1.14	0.03	− 6.49	2900	7.81	9	0.55	Yes	Yes	No	No
Swelling	1.66	0.03	8.09	2912	22.31	9	0.01	Yes	No	Yes	No
*Function*											
Clothes	− 1.07	0.04	− 3.22	2400	5.70	8	0.68	Yes	Yes	No	No
Wash hair	− 1.07	0.04	− 5.81	2383	9.93	8	0.27	No	No	Yes	No
Buttons	− 0.53	0.04	1.52	2393	1.89	8	0.98	No	No	Yes	Yes
Hold phone	− 0.47	0.04	0.42	2395	1.62	8	0.99	No	No	Yes	No
Reach across	− 0.43	0.04	− 0.83	2388	3.81	8	0.87	Yes	Yes	No	No
Grip handle	− 0.37	0.04	− 4.29	2380	7.32	8	0.50	No	No	No	No
Hold book	− 0.04	0.04	− 2.28	2373	3.22	8	0.92	Yes	Yes	No	No
Use hands	− 0.04	0.04	0.35	2383	2.79	8	0.95	No	No	No	No
Reach overhead	0.52	0.03	5.99	2401	7.64	8	0.47	Yes	Yes	No	No
Hold groceries	0.96	0.03	1.58	2395	1.83	8	0.99	Yes	Yes	No	No
Do chores	1.04	0.03	− 3.04	2399	7.45	8	0.49	Yes	Yes	No	No
Move arm	1.50	0.03	− 3.26	2383	4.31	8	0.83	No	No	No	No
*Appearance*											
People seeing	− 0.98	0.04	1.14	2412	8.50	9	0.49	Yes	Yes	No	No
Long-sleeved shirt	− 0.90	0.04	3.66	2418	6.68	9	0.67	Yes	Yes	No	No
Dress to hide	− 0.64	0.04	1.94	2411	2.90	9	0.97	No	No	No	No
Photos	− 0.37	0.04	− 3.64	2399	5.23	9	0.81	Yes	Yes	No	No
Noticeable	− 0.16	0.04	− 5.85	2421	6.64	9	0.67	No	No	Yes	−
Size	0.33	0.04	− 1.95	2430	3.92	9	0.92	Yes	Yes	No	No
Certain clothes	0.42	0.03	− 2.42	2424	3.99	9	0.91	No	No	No	No
Clothes fit	0.49	0.04	− 0.04	2416	4.50	9	0.88	No	No	Yes	Yes
Symmetry	0.57	0.04	− 4.85	2418	9.19	9	0.42	No	No	No	No
Sleeveless shirt	1.23	0.04	− 4.24	2426	10.57	9	0.31	No	No	No	No
*Psychological*											
Desperate	− 1.38	0.06	− 7.41	2264	15.81	8	0.05	Yes	No	No	No
Hopeless	− 0.85	0.05	− 6.13	2271.	13.11	8	0.11	Yes	No	No	No
Angry	− 0.52	0.04	− 4.63	2280	7.70	8	0.46	Yes	No	No	No
Depressed	− 0.39	0.04	− 4.85	2275	8.33	8	0.40	No	No	Yes	No
Stressed	− 0.16	0.04	− 5.71	2267	10.94	8	0.21	Yes	Yes	No	No
Afraid	− 0.08	0.04	− 2.66	2271	3.79	8	0.88	Yes	Yes	Yes	No
Anxious	− 0.03	0.04	− 0.98	2275	2.67	8	0.95	No	No	Yes	No
Fed-up	− 0.01	0.04	− 5.17	2277	8.19	8	0.42	No	No	No	No
Unattractive	0.66	0.04	4.86	2270	8.08	8	0.43	Yes	Yes	No	No
Worried	0.85	0.04	1.54	2276	2.53	8	0.96	Yes	No	No	No
Irritated	0.88	0.04	4.67	2277	7.49	8	0.48	Yes	Yes	No	No
Frustrated	1.03	0.04	− 0.18	2276	10.64	8	0.22	Yes	Yes	Yes	No
*Information*											
What it is	− 0.99	0.04	2.18	1325	12.47	8	0.13	No	No	No	No
Care for it	− 0.85	0.04	− 3.56	1318	10.64	8.	0.22	Yes	Yes	Yes	No
Avoid infections	− 0.40	0.04	3.31	1321	3.86	8	0.87	No	No	No	No
How its treated	0.07	0.04	− 4.87	1320	12.96	8	0.11	No	No	No	No
How to monitor	0.08	0.04	− 2.04	1319	6.21	8	0.62	No	No	No	No
Possibility	0.17	0.04	5.69	1322.	12.04	8	0.15	No	No	Yes	Yes
Healthcare team	0.51	0.04	0.22	1318	3.26	8	0.92	No	No	No	No
How it feels	0.60	0.04	− 3.12	1319	9.91	8	0.27	No	No	No	No
Impact on life	0.82	0.04	− 2.89	1321	12.54	8	0.13	No	No	No	No
*Sleeve*											
Fits	− 0.45	0.03	− 4.81	1935	8.68	9	0.47	No	No	No	No
Swelling	− 0.31	0.03	2.92	1950	3.28	9	0.95	Yes	Yes	No	No
Looks	− 0.26	0.03	1.08	1946.	4.01	9	0.91	Yes	Yes	No	No
Skin	− 0.17	0.03	4.01	1945	4.82	9	0.85	No	No	No	No
Put on	− 0.14	0.03	4.35	1953	3.75	9	0.93	No	No	Yes	No
Active	0.01	0.03	− 0.48	1942	4.66	9	0.86	Yes	Yes	No	No
Long wear	0.12	0.03	− 3.91	1938	9.01	9	0.44	Yes	Yes	No	No
Comfortable	0.15	0.03	− 4.66	1959	12.26	9	0.20	No	No	No	No
Dressed	0.38	0.03	− 1.45	1941	4.94	9.	0.84	No	No	No	No
Clothes fit	0.69	0.03	0.36	1945	4.39	9	0.88	Yes	Yes	No	No

SE = standard error; *df* = degrees of freedom; *χ*^2^ = chi-square

## Appendix 4

**Table:**

	None *N* = 418		Mild *N* = 1346		Moderate *N* = 1070		Severe *N* = 356	
	*N*	%	*N*	%	*N*	%	*N*	%
*a. Characteristics of the subgroups—self-reported severity of arm swelling* (*N* = 3190)								
Country								
Denmark	334	79.9	1165	86.6	990	92.5	337	94.7
USA	84	20.1	181	13.4	80	7.5	19	5.3
Age groups (years)								
≤ 49	33	7.9	153	11.4	93	8.7	31	11.5
50–59	86	20.6	379	28.1	274	25.6	109	30.6
60–69	161	38.5	429	31.9	345	32.2	97	27.3
≥ 70	138	33.0	385	28.6	358	33.5	109	30.6
BMI (kg/m^2^)								
Under/normal weight (< 25)	180	43.1	558	41.5	377	35.2	98	27.5
Overweight (25–29)	143	34.2	461	34.2	378	35.3	115	32.3
Obese (≥30)	94	22.5	325	24.1	310	29.0	140	39.3
Missing	1	0.2	2	0.2	5	0.5	3	0.9
Ethnicity								
Caucasian	370	88.5	1195	88.8	914	85.4	303	85.1
Other	48	11.5	151	11.2	156	14.6	53	14.9
Time since lymphedema diagnosis								
≤ 4	123	29.4	475	35.3	310	29.0	82	23.0
5–9	142	34.0	489	36.3	402	37.5	135	38.0
≥ 10	153	36.6	382	28.4	358	33.5	139	39.0
Lymphedema laterality								
Unilateral	413	98.8	1322	98.2	1044	97.6	345	96.9
Bilateral	5	1.2	24	1.8	26	2.4	11	3.1

	None *N* = 335		Minor *N* = 2108		Major *N* = 779	
	*N*	%	*N*	%	*N*	%
*b. Characteristics of the subgroups—based on having a problem with the arm(s) as a result of breast cancer and/or its treatment* (*N* = 3222)						
Country						
Denmark	302	90.1	1865	88.5	691	88.7
USA	33	9.9	243	11.5	88	11.3
Age groups (years)						
≤ 49	21	6.3	211	10.0	90	11.6
50–59	57	17.0	564	26.8	233	29.9
60–69	105	31.3	695	33.0	237	30.4
≥ 70	152	45.4	638	30.2	219	28.1
BMI (kg/m^2^)						
Under/normal weight (< 25)	152	45.4	834	39.6	240	30.8
Overweight (25–29)	114	34.0	734	34.8	259	33.3
Obese (≥30)	67	20.0	534	25.3	276	35.4
Missing	2	0.6	6	0.3	4	0.5
Ethnicity						
Caucasian	292	87.2	1844	87.5	670	86.0
Other	43	12.8	264	12.5	109	14.0
Time since lymphedema diagnosis						
≤ 4	94	28.1	683	32.4	221	28.4
5-9	132	39.4	774	36.7	277	35.6
≥ 10	109	32.5	651	30.9	281	36.0
Lymphedema laterality						
Unilateral	330	98.5	2080	98.7	746	95.8
Bilateral	5	1.5	28	1.3	33	4.2

	No sleeve *N* = 940		Sleeve *N* = 2282	
	*N*	%	*N*	%
*c. Characteristics of the subgroups—based on whether participant wore a compression sleeve in the past 12 months* (*N* = 3222)				
Country				
Denmark	807	85.9	2051	89.9
USA	133	14.1	231	10.1
Age groups (years)				
≤ 49	83	8.8	239	10.5
50–59	226	24.0	628	27.5
60–69	319	34.0	718	31.5
≥ 70	312	33.2	697	30.5
BMI (kg/m^2^)				
Under/normal weight (< 25)	350	37.2	876	38.4
Overweight (25–29)	328	34.9	779	34.1
Obese (≥ 30)	260	27.7	617	27.1
Missing	2	0.2	10	0.4
Ethnicity				
Caucasian	811	86.3	1995	87.4
Other	129	13.7	287	12.6
Time since lymphedema diagnosis				
≤ 4	217	23.1	781	34.2
5–9	350	37.2	833	36.5
≥ 10	373	39.7	668	29.3
Lymphedema laterality				
Unilateral	913	97.1	2243	98.3
Bilateral	27	2.9	39	1.7
